# Identification of fragments binding to SARS-CoV-2 nsp10 reveals ligand-binding sites in conserved interfaces between nsp10 and nsp14/nsp16†

**DOI:** 10.1039/d1cb00135c

**Published:** 2021-10-06

**Authors:** Frank Kozielski, Céleste Sele, Vladimir O. Talibov, Jiaqi Lou, Danni Dong, Qian Wang, Xinyue Shi, Maria Nyblom, Annika Rogstam, Tobias Krojer, Zoë Fisher, Wolfgang Knecht

**Affiliations:** School of Pharmacy, University College London 29-39 Brunswick Square London WC1N 1AX UK; Department of Biology & Lund Protein Production Platform, Lund University Sölvegatan 35 22362 Lund Sweden wolfgang.knecht@biol.lu.se +46 46 2227785; BioMAX beamline, MAX IV Laboratory Fotongatan 2 22484 Lund Sweden; European Spallation Source ERIC P.O. Box 176 22100 Lund Sweden zoe.fisher@ess.eu +46 721792250

## Abstract

Since the emergence of SARS-CoV-2 in 2019, Covid-19 has developed into a serious threat to our health, social and economic systems. Although vaccines have been developed in a tour-de-force and are now increasingly available, repurposing of existing drugs has been less successful. There is a clear need to develop new drugs against SARS-CoV-2 that can also be used against future coronavirus infections. Non-structural protein 10 (nsp10) is a conserved stimulator of two enzymes crucial for viral replication, nsp14 and nsp16, exhibiting exoribonuclease and methyltransferase activities. Interfering with RNA proofreading or RNA cap formation represents intervention strategies to inhibit replication. We applied fragment-based screening using nano differential scanning fluorometry and X-ray crystallography to identify ligands targeting SARS-CoV-2 nsp10. We identified four fragments located in two distinct sites: one can be modelled to where it would be located in the nsp14–nsp10 complex interface and the other in the nsp16–nsp10 complex interface. Microscale thermophoresis (MST) experiments were used to quantify fragment affinities for nsp10. Additionally, we showed by MST that the interaction by nsp14 and 10 is weak and thereby that complex formation could be disrupted by small molecules. The fragments will serve as starting points for the development of more potent analogues using fragment growing techniques and structure-based drug design.

## Abstract 2

By fragment screening using X-ray crystallography we identified four ligands revealing ligand-binding sites in interfaces between SARS-CoV-2 nsp10 and nsp14/nsp16. The nsp14/10 interaction is weak and therefore could be disrupted by small molecules.

## Graphical abstract

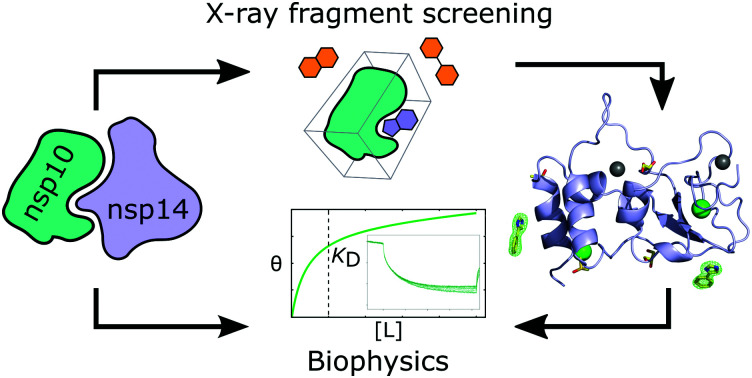

## Introduction

The ongoing Coronavirus disease 19 (Covid-19) pandemic, caused by Severe Acute Respiratory Syndrome Coronavirus-2 (SARS-CoV-2) is a threat to our health, social and economic systems.

Vaccine development is currently a cornerstone of managing the ongoing pandemic and several vaccines have been approved and many more are being developed (WHO, https://www.who.int/emergencies/diseases/novel-coronavirus-2019/covid-19-vaccines/advice and https://www.who.int/publications/m/item/draft-landscape-of-covid-19-candidate-vaccines, accessed 2021-09-24). However, the duration of protection by vaccination is currently unknown and booster vaccinations may be necessary in the near future. Another major issue is the emergence of SARS-CoV-2 variants, some of which may have the potential to lead to higher infection rates and/or more severe illness^1^ or may even reduce the effectiveness of current vaccines (ECDC, https://www.ecdc.europa.eu/en/covid-19/variants-concern, accessed 2021-09-24). So far, the repurposing of existing drugs has not been as successful as vaccine development. Only one established medication, dexamethasone, has been shown to reduce the mortality of Covid-19 patients on respiratory support.^2^

There is a need to develop specific drugs against SARS-CoV-2 and potential future outbreaks to complement the use of vaccines. Having drugs available that target SARS-CoV-2 are important as a second line of defense if for example vaccination cannot happen or is ineffective long-term. The development of drugs that target SARS-CoV-2 is also of utmost importance for people with reduced immune function, for whom vaccines may not be effective or suitable as for the wider population and if emerging viral variants suddenly compromise vaccine efficacy.

One of the targets to possibly combat SARS-CoV-2 is nonstructural protein (nsp) 10, which forms a complex with two other viral nsps, nsp14 and nsp16. Coronaviruses (CoVs) stand out among RNA viruses because of their low mutation rate despite their relatively large genomes.^3^ This is because nsp14, a bifunctional enzyme, plays a vital role in viral replication.^4^ At its N-terminus it carries a 3′-5′ exoribonuclease (ExoN) activity that excises nucleotide mismatches at its RNA 3′-end, presumably limiting the efficacy of nucleoside analogue-based drugs such as ribavirin and remdesivir against CoVs.^3^ In CoVs, a capping machinery is crucial and ensures that the viral RNA escapes destruction by the host cell. Nsp14 also carries an N7-methyltransferase (N7-MTase) function at its C-terminus, one of two MTase activities required. The other is provided by nsp16 that has 2′-O-MTase activity^5^ to complete RNA cap formation.

In SARS, nsp10 binds to the N-terminus of nsp14 and activates the full potential of the ExoN activity but does not seem to be required for the stimulation of the N7-MTase.^4,6^ We hypothesise that approaches to suppress viral replication include inhibiting the ExoN activity of nsp14, the MTase functions of nsp14 and 16, or inhibiting the stimulating action of nsp10 by blocking binding to either nsp14 or 16 with small molecules. As nsp10 is not found in host cells, targeting nsp10 and thereby indirectly nsp14 and nsp16, provides an exclusive and targeted strategy to prevent SARS-CoV-2 replication.

The crystal structure of SARS-CoV-2 nsp10 has been determined by several groups in complex with nsp16 or the ExoN domain of nsp14. SARS-CoV-2 nsp10 in complex with nsp16 was reported early on.^7–9^ While writing this manuscript, the crystal structure of a catalytically inactive SARS-CoV-2 ExoN mutated in an active site residue in complex with nsp10 became available^10^ followed by nsp10 in complex with active ExoN.^11^ The later work concluded a variable role of nsp10 α1 helix in engagement of nsp14 or nsp16, interacting with nsp14, but not nsp16. However, nsp14 and nsp16 interact largely with an overlapping area of nsp10.

We recently determined the crystal structure of the unbound form of SARS-CoV-2 nsp10 to 1.55 Å resolution and described its close structural relationship to SARS nsp10.^12^ The high resolution and favourable crystal system parameters open up the opportunity to discover nsp10-targeting ligands that could interfere with complex formation by using fragment-based screening *via* X-ray crystallography. The aim of this study was to identify fragments binding to SARS-CoV-2 nsp10 as a starting point for structure-based drug design and as chemical probes to describe druggable binding pockets in nsp10. As nsp10 forms complexes with at least two other non-structural SARS-CoV-2 proteins, nsp14 and nsp16, we also aimed at establishing an assay that would allow probing and quantification of the interaction with one of its interaction partners, nsp14. This can later also be used in characterizing compound-mediated interruption of such interactions.

## Results and discussion

To identify fragments interacting with nsp10, we employed two orthogonal assays in parallel, X-ray based fragment screening (XFS) and thermal shift assay (TSA).

### XFS

We used 107 of the 110 fragments available (Table S1, ESI†) at the FragMAX facility.^13^ After soaking the crystals, diffraction data sets of the crystals were collected at BioMAX beamline of MAX IV.

**Fig. 1:**
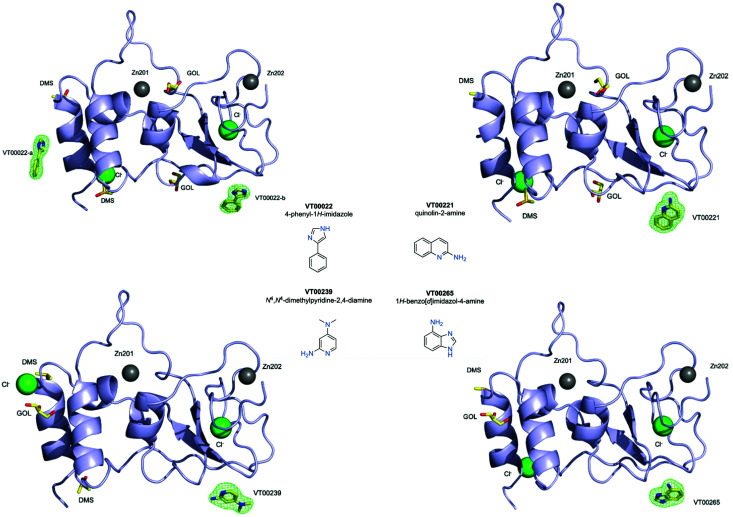
Fragment hits bounds to nsp10 from the FragMAX library. Crystal structures of nsp10 in complex with four fragment hits. Nsp10 is shown in lilac cartoon, structural zinc atoms are shown as grey spheres, chloride ions as green spheres, and fragments and other small molecule ligands are shown in yellow ball-and-stick and are as labelled. *mF*_o_ − *DF*_c_ electron density omit maps are shown in green mesh and are contoured at 3.0*σ*. The chemical structures and systematic names of the four fragment hits targeting SARS-CoV-2 nsp10 are shown in the middle.

Initial attempts to analyse obtained datasets using PanDDA software^14^ were not successful. Therefore, screening results were assessed by means of inspection of *mF*_o_ − *DF*_c_ difference density maps. We were able to clearly identify four bound fragments from the FragMAX library^13^ which corresponds to a hit rate of 3.8% (Table S1, ESI†). Data collection and refinement statistics for obtained nsp10–fragment complexes are summarised in Table 1 and the chemical structures of the fragment hits are shown in Fig. 1. We found that the four fragments (Table 1) bound to nsp10 occupy two different binding sites (Fig. 1, upper left). All fragments had very strong difference peaks in the *mF*_o_ − *DF*_c_ omit maps and also show excellent 2*mF*_o_ − *DF*_c_ electron density and refined with full occupancy. Three of the fragments bound to the same site, while one fragment bound to two sites (Fig. 1) in nsp10. However, the only two fragments found to stabilize nsp10 (VT00029 & VT00213) in the TSA did not appear as hits in crystallographic screening.

**Table 1:** Data collection, data processing, and model refinement statistics for four nsp10–fragment complexes from SARS CoV-2. Data in parenthesis correspond to the highest resolution shell. Refinement statistics were calculated using MolProbity server^15^

PDB ID	Nsp10–VT00022	Nsp10–VT00221	Nsp10–VT00239	Nsp10–VT00265
	7ORR	7ORU	7ORV	7ORW
Data reduction				
Wavelength [Å]	0.979	0.979	0.979	0.979
Resolution range [Å]	74.87–1.79 (1.83–1.79)	37.75–1.67 (1.70–1.67)	28.66–1.95 (2.00–1.95)	76.21–1.95 (2.00–1.95)
Space group	*I*2_1_3	*I*2_1_3	*I*2_1_3	*I*2_1_3
Unit cell parameters (Å, °)	*a* = *b* = *c* = 105.88; *α* = *β* = *γ* = 90	*a* = *b* = *c* = 106.78; *α* = *β* = *γ* = 90	*a* = *b* = *c* = 107.24; *α* = *β* = *γ* = 90	*a* = *b* = *c* = 107.77; *α* = *β* = *γ* = 90
Total reflections	202 442 (6669)	676 492 (12 343)	148 494 (10 876)	129 317 (8387)
Unique reflections	18 629 (1113)	23 619 (1205)	15 070 (1060)	15 364 (1038)
Multiplicity	10.9 (6.0)	28.6 (10.2)	9.9 (10.3)	8.4 (7.7)
Completeness [%]	99.3 (100.0)	100.0 (99.7)	99.8 (100.0)	100.0 (100.0)
Mean *I*/sigma(*I*)	24.3 (1.5)	27.3 (1.3)	14.8 (1.6)	13.5 (1.5)
*R*_meas_	0.049 (1.377)	0.072 (1.653)	0.077 (1.068)	0.08 (1.42)
*R*_pim_	0.014 (0.556)	0.012 (0.503)	0.024 (0.333)	0.028 (0.506)
CC_1/2_	1.0 (0.572)	1.0 (0.506)	0.999 (0.752)	0.998 (0.567)

Model refinement				
*R*_cryst_/*R*_free_ [%]	17.2 (28.1)/18.4 (26.1)	16.1 (28.0)/17.2 (28.9)	17.0 (27.7)/20.2 (28.9)	17.4 (26.9)/20.1 (28.8)
Total no. of non-hydrogen atoms (protein)	1053	1098	1044	1026
No. of protein/ligand/solvent atoms	915/46/92	913/29/156	921/29/94	916/24/86
Average *B*-factor/protein/ligands/solvent	45.5/44.3/57.1/51	35.7/33.9/42.5/45.2	52.3/51.8/61.7/54.8	52.3/51.7/65.4/55.1
RMSD (bonds, angles)	0.014/1.7	0.013/1.7	0.014/1.7	0.014/1.7
Ramachandran favored/allowed/outliers/rotamer outliers [%]	97.5/2.5/0.0/0.0	97.5/2.5/0.0/0.0	97.5/2.5/0.0/0.97	98.4/1.6/0.0/0.98
Clashscore	1.06	1.08	1.62	0.55

### Description of nsp10–fragment interactions

**Fig. 2:**
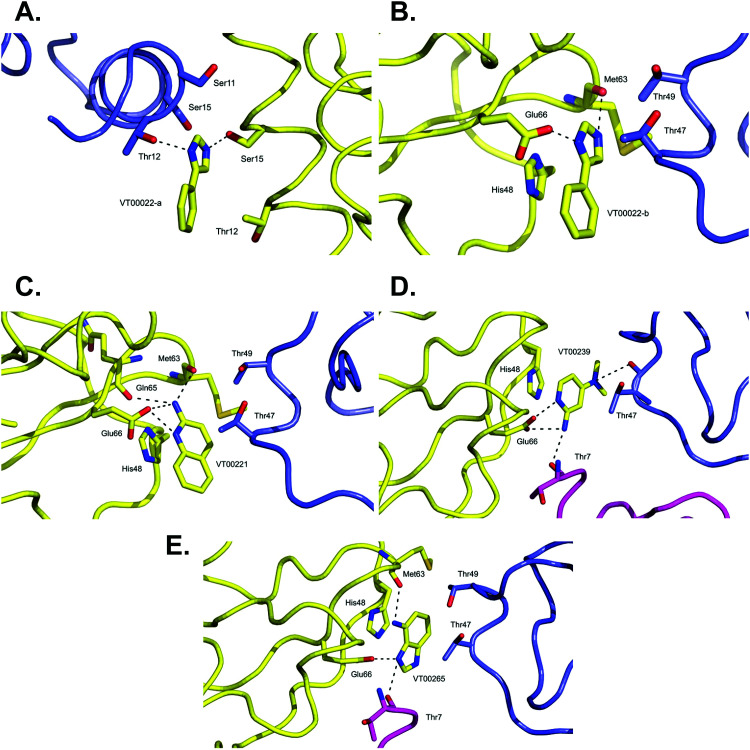
Close-up view of ligand binding sites of SARS-CoV-2 nsp10 for identified fragments. The protein is shown in yellow ribbon and with symmetry related chains in lilac and magenta. The relevant amino acid side chains or main chain components are shown as sticks where relevant. Hydrogen bonds identified through Ligplot^16^ are indicated as black dashed lines. VT00022 binding in the (A) nsp14–nsp10 and (B) nsp16–nsp10 interfaces. The other three fragments (C) VT00221, (D) VT00239 and (E) VT00265 all bind in the nsp16–nsp10 interface.

VT00022 binds to two ligand binding sites on nsp10 (Fig. 2A and B). In the first pocket the 4-phenyl group establishes a hydrophobic interaction with Thr12, and also with Ser11 and Ser15 from the symmetry related molecule. The 1*H*-imidazole moiety establishes a hydrogen bond interaction with the side chain of Ser15 (∼2.8 Å) and Thr12 (∼3.0 Å) from the symmetry related molecule (Fig. 2A). Interactions in the second pocket are more numerous with a π-stacking interaction with His48 (∼3.8 Å), and hydrogen bonds between the imidazole moiety and Glu66 (∼2.6 Å) and the main chain carbonyl of Met63 (∼2.8 Å). In addition, there are hydrophobic interactions with Thr47 and Thr49 from the symmetry related molecule (Fig. 2B).

The main characteristic of the binding interactions between nsp10 and VT00221 is the elevated number of hydrogen bond interactions (Fig. 2C). These are formed between the 2-amine and the aromatic nitrogen atom of the quinoline group with the side chain oxygen of Glu66 (∼2.9 Å), and the main chain carbonyl atoms of Met63 (∼3.0 Å) and Gln65 (∼3.1 Å). There is also a π-stacking interaction between His48 (∼3.4 Å) and hydrophobic interactions with symmetry related residues Thr47, and Thr49 (Fig. 2C).

In VT00239, the molecule engages in hydrogen bond interactions with the side chain of Glu66 (∼2.7 Å), and two additional weak hydrogen bonds to symmetry related chains: Thr7 (∼3.5 Å) and Thr47 (∼3.2 Å). The ring system makes a π-stack with His48 (∼3.5 Å) and a potential hydrophobic interaction with Thr47 of a symmetry related molecule (Fig. 2D).

In VT00265, the imidazole moiety makes two hydrogen bonds, one with the side chain of Glu66 (∼2.7 Å) and the other with the carbonyl oxygen of Thr7 (∼3.2 Å) from a symmetry related molecule. The 4-amine substituent at N1 makes a hydrogen bond with the carbonyl oxygen of Met63 (∼3.0 Å). Like the other fragment hits, VT000265 also makes a π-stack with His48 (∼3.5 Å). There are also possible hydrophobic interactions with Thr47 and Thr49 from a symmetry related molecule (Fig. 2E). In summary, residues Thr7, Thr47, Thr49, His48, Met63 and Glu66 of nsp10 are key residues involved in binding of fragments through both hydrophobic and charged interactions. Interactions between fragments and symmetry related molecules in the crystal may further stabilize the weak binding and are probably favoured in the crystalline form.

A shared key feature of hits binding in the nsp16–nsp10 interface is the presence of two nitrogen atoms, separated by one or maximal two carbon atoms, which allow establishing hydrogen bond interactions with either Glu66 alone or with Met63 and Glu66, revealing shared chemical requirements for binding. It is also noteworthy that the number of hydrogen bond and hydrophobic interactions between residues of nsp10 and the fragment hits does not correlate with their measured *K*_d_ values.

### TSA with fragments using nanoDSF and further characterisation using microscale thermophoresis (MST) to determine affinities

The 110 fragments (Table S1, ESI†) from the library of the FragMAX platform^13^ were also tested for their effects on nsp10 in a thermal shift assay (Fig. S1, ESI†). The outcome of this assay can be a positive or negative *T*_m_ shift, no shift at all, or a wide variety of atypical thermal denaturation curves. For an in depth discussion of those see ref. 17.

Only two fragments (VT00029 & VT00213) were found to have an equal or higher *T*_m_ than the average + 3 × SD (= 47.8 °C) in both runs, while many compounds showed a decrease in *T*_m_. Also, many atypical curves that did not allow us to determine *T*_m_ or a wide spread between the two experiments were observed (Table S1, ESI†). The measured *T*_m_ values of the fragment hits from XFS all showed negative shifts or did not provide standard melting curves in TSA experiments (Table 2). *Vice versa*, the two fragments stabilizing nsp10 by TSA were not detected in crystal soaking experiments. We conclude that at the current stage of the project, TSA is not a suitable assay to select candidate fragments for co-crystallizability with nsp10, due to poor assay quality and because we lack a tool compound, therefore we do not yet know if stabilisation or de-stabilisation by a small molecule is the desired determinant of co-crystallizability or mode of action of the compound. This exemplifies the advantage of XFS over TSA as a screening approach, as molecular details of interactions are instantly visible and can be exploited for subsequent elaboration of screening hits.

**Table 2:** Summary of XFS, MST and TSA results for nsp10 targeting fragment hits and calculated properties of the fragments. The Δ*T*_m_ values of nsp10 in the presence of fragments obtained from nanoDSF and fragment affinities for nsp10 as determined by MST are given. Molecular weight, MW; polar surface areas, PSA; calculated logP, clogP; HBA, hydrogen bond acceptor; HBD, hydrogen bond donor; MolLogS, calculated solubility; lifc, ligand-induced fluorescence change. TSA experiments were conducted in duplicate, whereas MST experiments were carried out in triplicate and are therefore presented individually or as average ± SD. The average *T*_m_ of nsp10 without any ligand under the same assay conditions was determined to be 46.6 ± 0.4 °C (*n* = 11)

Fragment ID	Binding site	MST nsp10 *K*_d_ [mM]	TSA nsp10 Δ*T*_m_ [°C]	MW [Da]	clogP	HBA and HBD	tPSA [Å^2^]	MolLogS [Log (moles L^−1^)]
VT00022	1 & 2	>20 mM	Atypical curve	144.07	1.35	1/1	24.39	−1.60
VT00221	2	7.4 ± 3.1	−2.6 and −2.3	144.18	0.80	1/2	38.38	−2.21
VT00239	2	1.9 ± 0.7	−3.6 and −3.5	137.19	−0.42	1/2	41.62	−1.05
VT00265	2	lifc	0 and −1.3	133.15	−0.38	1/3	50.41	−1.10

To further characterise the interaction between nsp10 and our novel fragment hits we chose MST as an orthogonal biophysical technique. We subsequently established and optimised MST assays for measuring the affinity of the fragments for nsp10, quantified by *K*_d_ values. VT00022 is a very weak binder, and although clear density is visible in the difference map (Fig. 1, upper left), we could not determine its apparent *K*_d_ value under the experimental conditions used. VT00221 and VT00239 show *K*_d_ values of 7.4 ± 3.1 and 1.9 ± 0.7 mM, in a range expected for fragment hits. Because MST is conducted in solution when the protein is monomeric and does not have the extra interactions with symmetry mates present in the crystal, the measured *K*_d_ values proof that fragment binding does not require the interactions from the symmetry mates.

The measured *K*_d_ value for VT00265 was below 20 μM, being too low for a typical fragment screening hit. Close inspection of the experimental data indicated assay interference for this ligand. We observed a significant ligand-induced fluorescence change when the ligand concentration increases. The fluorescence count of the sample with 5 mM VT00265 was approximately 10-fold less than the sample with 300 nM fragment. We conducted an EDTA/control-peptide (ECP) test to determine whether the observed ligand-induced fluorescence change is caused by the protein–ligand interaction or by unspecific effects such as aggregation, adsorption to the labware, ligand interaction with the His-tag or the RED-tris-NTA labelling dye. The EDTA test showed that nsp10 aggregated at high fragment concentrations causing an 8-fold decrease in fluorescence count. Moreover, the control-peptide test showed that VT00265 caused a significant ligand-induced fluorescence change with 20-fold decrease in fluorescence, compared to the reference sample. These results indicate that the high VT00265 concentration causes nsp10 aggregation, but the main reason for the assay interference was quenching of the fluorescence signal. Therefore, another technique than TSA or MST, should be employed for this particular fragment.

All fragment hits comply to the Rules of Three (RO3) with molecular weights between 133 and 144 Da, clogP values lower than three and less or equal to three hydrogen bond donors and acceptors. The polar surface area was calculated to be between 24 and 50 Å^2^, and the calculated solubility MolLogS was good for all fragments with the exception of VT00221, which showed an approximately 3- to 11-fold reduced solubility compared to the other three hits.

### Nsp10 targeting fragment hits reveal novel binding sites located in nsp14 and nsp16 interfaces with nsp10

**Fig. 3:**
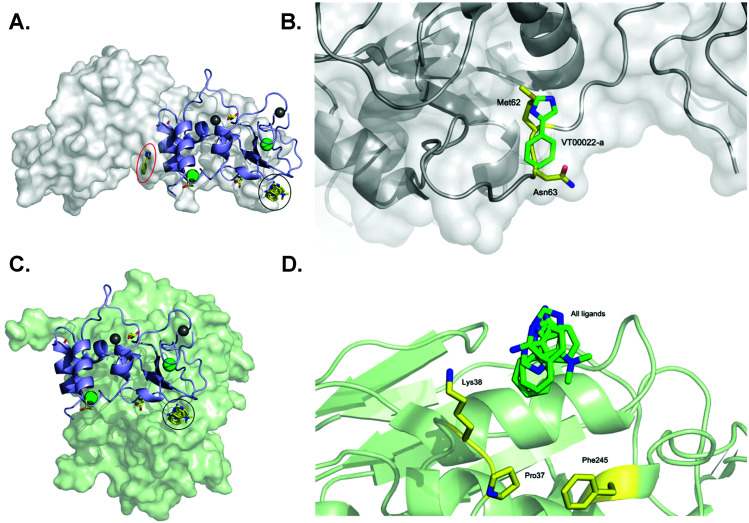
SARS-CoV-2 nsp10 and all four fragment hits modelled onto either nsp10–nsp14 or nsp10–nsp16 complexes. In all panels, nsp10 is shown in lilac cartoon, nsp14 is shown in grey as either spheres or cartoon, and nsp16 is shown in pale green as either spheres or cartoon. VT00022-a located in the nsp10–nsp14 interface is circled in red (binding site 1) whereas VT00022-b and the other three ligands located in binding site 2 are superimposed and circled in black. (A) The SARS-CoV-2 ExoN-nsp10 complex (PDB ID 7MC5) with ligands superimposed. (B) Magnification of the VT00022 binding location overlapping with residues of the nsp14 ExoN domain. (C) The SARS-CoV-2 nsp10–nsp16 complex (PDB ID 7LW4) with ligands superimposed, located in proximity to the nsp16–nsp10 interface. (D) Magnification of the potential binding site on nsp16.

One of our aims was to identify fragment hits binding to nsp10 and explain these in the context of nsp10's biological function. To clarify the importance of these novel ligand binding pockets on nsp10, we modelled the nsp10–fragment complexes determined here with crystal structures of nsp10 bound to either nsp14 or nsp16. Interestingly, one of the binding sites on nsp10 for VT00022 is located exactly in the interface between nsp14 and nsp10 (Fig. 3A). A magnification of the binding site reveals that VT00022 would directly clash with residues Met62 and Asn63 of nsp14 (Fig. 3B). Similarly interesting, the second ligand binding site identified is occupied by all four fragment hits and is located in the interface between nsp10 and nsp16, its second binding partner (Fig. 3C). These fragments could interfere with the formation of the nsp16–nsp10 complex as they are located directly in the interface, within 5–9 Å of residues Pro37, Lys38, and Phe245 of nsp16 (Fig. 3D). Potentially these hits could be further developed into protein–protein interaction inhibitors preventing the formation of the complex. Currently, we do not know if there are additional ligand binding sites on nsp10, which may be identified by screening of larger fragment libraries. Additionally, we do not know if the identification of these two binding sites in the two protein–protein interfaces is a coincidence or could be favoured by the complexes. Currently the regulation of interactions between nsp10 and nsp14 as well as nsp16, the association and the dissociation mechanisms, are unknown.

### SARS-CoV-2 nsp14 and nsp10 form a weak affinity complex

**Fig. 4:**
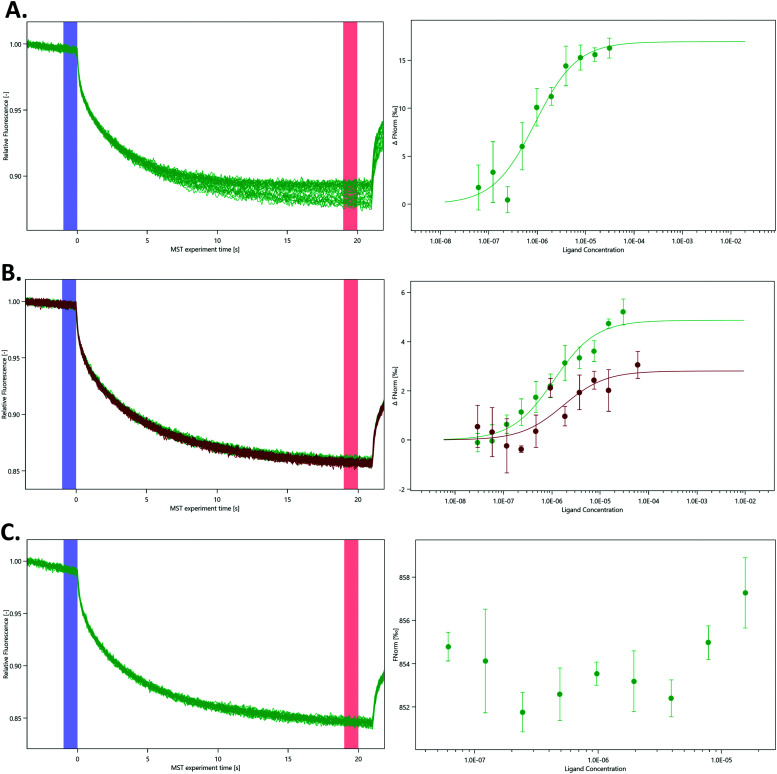
Quantification of the interaction between various nsp14 domains and nsp10 using MST. MST traces (left panels) show changes in fluorescence upon activation and deactivation of the IR laser. Dose–response curves (right panels) display changes in the ratio between the fluorescence after 5 s MST on time and the fluorescence before the activation of the IR laser under different nsp10 concentrations. (A) N-Terminal ExoN domain, (B) full-length nsp14 in the absence (green) and presence of 3% deuterated DMSO (red) and (C) the C-terminal N7-MTase domain with nsp10. As there was no binding event between the N7-MTase domain and nsp10, the Fnorm figure is presented as provided by the MO. Affinity Analysis software. All experiments were conducted at least in triplicate.

In the absence of affinity (and at the time of writing also structural) data for the SARS-CoV-2 nsp14–nsp10 complex we decided to probe the interaction using various nsp14 constructs and nsp10 *via* MST (Fig. 4). We purified three SARS-CoV-2 nsp14 constructs containing the His-SUMO-tag and labelled the proteins with second generation dye. The interaction between these tagged nsp14 constructs and full-length nsp10 was subsequently quantified by preparing dilutions series of nsp10 and incubating those individually with the three nsp14 constructs. For the ExoN domain, we measured a *K*_d_ value of 0.9 ± 0.3 μM, indicating that the interaction between nsp14 and nsp10 is not very strong (Fig. 4A). Similarly, for full-length nsp14, we determined a *K*_d_ value of 1.1 ± 0.9 μM and a *K*_d_ value of 1.4 ± 0.3 μM in the absence or presence of 3% deuterated DMSO, respectively. This is in a similar range as that observed for ExoN. These results also indicate that the protein–protein interacting assay can be performed in the presence of inhibitor stocks prepared in DMSO (Fig. 4B). Finally, we also tested for a potential interaction between the nsp14 N7-MTase domain and nsp10, but did not observe any interacting between these two proteins under similar experimental conditions (Fig. 4C) thereby also excluding any artefacts due to the presence of the N-terminal tag. Recently we became aware of work showing the interaction of nsp10 and nsp14 from SARS-CoV-2 using SPR.^18^ While the authors show an interaction between these two proteins, steady-state analysis of interaction curves did not allow to unambiguously quantify *K*_d_ values for the interaction from these experiments.^18^ MST therefore shows excellent potential to study and quantify the interaction between nsp14 and nsp10.

While we still lack structural data for the SARS-CoV-2 full-length nsp14–nsp10 complex, the structure for its SARS homologue has been previously determined^3,4^ confirming this type of arrangement deduced by our MST data in which only the ExoN domain but not the N7-MTase domain is involved in binding to nsp10. Owing to the very high protein sequence identify between SARS and SARS-CoV-2 nsp14,^18^ we hypothesise that the SARS-CoV-2 nsp14–nsp10 complex displays the same characteristics as observed for the SARS complex and that the affinity is relatively weak with *K*_d_ values in the low micromolar range. To the best of our knowledge, the work shown here is the first time the quantitative interaction between the two proteins is reported for coronaviruses.

## Conclusions

We identified and characterised the first interactions between fragments and SARS-CoV-2 nsp10. This work revealed their binding sites, both in terms of molecular detail and in the context of the protein–protein complexes nsp10 makes in the viral replication machinery. In addition, we have quantitatively shown that the nsp10–nsp14 complex is a relatively weak affinity complex. Crystallographic fragment screening with low complexity ligands rarely provides ready molecular probes for downstream applications, *e.g.* for study of nsp10–nsp14 or nsp10–nsp16 interfaces and nsp10 biology. However, we showed that nsp10 regions, involved in its interactions with other proteins, are targetable by small molecules. For further optimisation of the identified fragments, we are in the process to increase the affinity of the current hits using SAR-by-catalogue and structure-based design approaches. Due to the structures of the fragments and their location, we are presently restricted to mainly apply fragment-growing techniques in contrast to being able to use fragment linking. Currently, a larger fragment library is being screened to increase the number of starting points for subsequent chemical optimisation.

## Experimental

### Subcloning, expression and purification of nsp10 constructs

For XFS and TSA with nanoDSF a short SARS-CoV-2 nsp10 construct was expressed and purified as recently described.^12^

For the determination of *K*_d_ values for nsp10–fragment complexes by MST, it was purified as follows. Expression was done in *E. coli* BL21-CodonPlus (DE3)-RIPL competent cells (Agilent Technologies, Santa Clara, CA, USA) in Terrific Broth modified medium (Melford, Chelsworth, UK) supplemented with 50 μg ml^−1^ Kanamycin and 34 μg ml^−1^ Chloramphenicol. Cultures were incubated at 37 °C while shaking at 220 rpm until OD_600_ 0.6–1.0. Induction of protein expression was done with 1 mM isopropyl-β-d-thiogalactopyranoside (IPTG). Cultures were then incubated at 18 °C and shaken at 220 rpm for 24 h before the cells were harvested. Cell pellets were re-suspended in buffer A (50 mM sodium phosphate buffer (NaPO_4_) pH 8.0, 300 mM NaCl, 20 mM imidazole and 1 mM phenylmethylsulfonylfluoride (PMSF)), flash-frozen in liquid nitrogen and stored at −80 °C.

Cell pellets were thawed at room temperature and lysed by sonication. The cell lysate was centrifuged at 45 000 × *g* for 1 h at 4 °C and the supernatant was loaded into a 5 ml HisTrap FF crude column (Cytiva, Uppsala, Sweden) pre-equilibrated with buffer B (50 mM NaPO_4_ pH 8.0, 300 mM NaCl and 20 mM imidazole) followed by washing with 50 column volumes (CVs) of buffer B. SUMO-nsp10 was eluted with buffer C (50 mM NaPO_4_ pH 8.0, 300 mM NaCl and 250 mM imidazole). Samples containing SUMO-nsp10 were pooled. The purified SUMO-nsp10 was directly dialysed against 50 mM NaPO_4_ pH 8.0 and 150 mM NaCl buffer at 4 °C and concentrated to around 20 mg ml^−1^ for storage at −80 °C.

To study the interaction between nsp14 and nsp10, full-length nsp10 without affinity tag was prepared as follows. A codon-optimised DNA (Genscript, Leiden, Netherlands) insert for expression in *E. coli* coding for residues 1 to 139 of SARS-CoV-2 nsp10 was subcloned into the ppSUMO-2 vector using NcoI and XhoI restriction sites.^19^ Expression and purification was carried out as described above for the shorter nsp10 construct, but an additional purification step was added to remove the SUMO tag. The affinity tag was cleaved by His-tagged ULP-1 protease (pFGET19_Ulp1) and was a gift from Hideo Iwai (Addgene plasmid # 64697; http://n2t.net/addgene:64697; RRID:Addgene_64697). Cleavage was done in the presence of 1 mM dithiothreitol (DTT) while dialysing against 50 mM NaPO_4_ pH 8.0, 300 mM NaCl, 20 mM imidazole and 1 mM DTT for 18–20 h at 4 °C. The sample was purified through a second 5 ml HisTrap FF crude column. Samples containing full-length nsp10 were pooled and dialysed overnight at 4 °C against buffer D (50 mM Tris–HCl pH 8.0 and 150 mM NaCl). The sample was concentrated to 60 mg ml^−1^ for storage at −80 °C.

### Subcloning, expression and purification of nsp14 constructs

The cDNAs for the three nsp14 protein constructs, codon-optimised for *E. coli* expression, were obtained from Genscript. They were subcloned into the ppSUMO-2 vector using *NcoI* and *XhoI* restriction sites resulting in constructs coding for an N-terminal His-tag, followed by a SUMO tag, a ULP1 protease cleavage site, and the nsp14 cDNA. They represent full length nsp14, the N-terminal ExoN domain and the C-terminal N7-MTase domain and were delineated using the structure and sequence of the SARS nsp14–nsp10 complex.^4^ For the design of full-length SARS-CoV-2 nsp14, the protein sequence of SARS nsp14 was extracted from the crystal structure of the nsp14–nsp10 complex and aligned with the ORF1ab protein sequence of the Wuhan coronavirus sequence (GenBank: MN908947.3). SARS and SARS-CoV-2 nsp14 share 95.1% sequence identify and 3.4% strongly similar residues. This construct is called full-length nsp14 throughout the manuscript. The protein sequence covering the SARS-CoV-2 N-terminal ExoN domain was generated by using the structural information of SARS nsp14. The two domains of full-length nsp14 are well separated by a small antiparallel β-sheet. Based on this structural arrangement the construct containing residues 1 to 289, is named ExoN and the domain encompassing residues 282 to 527 is named the N7-MTase.

Expression experiments in a range of *E. coli* strains revealed distinct optimal expression conditions for each nsp14 construct, and that purification had to be optimized for each protein individually:

The expression of the nsp14 ExoN domain was carried out in *E. coli* BL21-Rosetta (DE3) (Merck KGaA, Darmstadt, Germany) using cell culture conditions as described for nsp10. Cell pellets were resuspended in 50 mM Tris–HCl pH 8.4, 300 mM NaCl, 10 mM imidazole, and 1 mM PMSF and lysed by sonication for 30 s for 10 rounds, with a 60 sec rest period in between. Cell debris was removed by centrifugation at 11 400 × *g* for 60 min at 4 °C. The supernatant was loaded onto a 5 ml HiTrap TALON crude column (Cytiva, Uppsala, Sweden) equilibrated in 50 mM Tris–HCl pH 8.4, 300 mM NaCl and 10 mM imidazole. The column was washed with 50 mM Tris–HCl pH 8.4, 300 mM NaCl and 20 mM imidazole, and bound protein was eluted with 50 mM Tris–HCl pH 8.4, 300 mM NaCl, and 200 mM imidazole. Fractions containing ExoN were pooled and dialysed against 50 mM Tris–HCl pH 8.4 and 300 mM NaCl at 4 °C and subsequently concentrated to 2.9 mg ml^−1^ and stored at −80 °C.

The expression of the nsp14 N7-MTase domain was done in *E. coli* BL21-CodonPlus (DE3)-RIPL competent cells using cell culture conditions as described for nsp10. The cell pellet was resuspended in 50 mM Tris–HCl pH 7.8, 300 mM NaCl, 10 mM imidazole, and 1 mM PMSF and the lysate was prepared by sonication and centrifugation as described above for ExoN. The supernatant was loaded onto a 5 ml HiTrap TALON column equilibrated in lysis buffer. The column was washed with 50 mM Tris–HCl pH 7.8, 300 mM NaCl, and 20 mM imidazole. The protein was eluted with 50 mM Tris–HCl pH 7.8, 300 mM NaCl, and 200 mM imidazole. Samples containing N7-MTase were pooled, concentrated and further separated on a HiLoad 16/600 Superdex 200 pg size exclusion column (Cytiva, Uppsala, Sweden) in 50 mM Tris–HCl pH 7.8 and 150 mM NaCl. Fractions containing N7-MTase were collected, concentrated to 5.9 mg ml^−1^ and stored at −80 °C.

Full-length SARS-CoV-2 nsp14 was expressed in *E. coli* TUNER (DE3) cells (Novagen, Darmstadt, Germany) using culture conditions as described above for nsp10, but without chloramphenicol. Cell pellets were resuspended in 50 mM Tris–HCl pH 8.4, 300 mM NaCl and 10 mM imidazole, and supplemented with 1 mM PMSF before being stored at −80 °C. The cells were thawed at room temperature and lysed as described above for ExoN. Cell debris was removed by centrifugation at 10 000 × *g* for 1 h at 4 °C. The supernatant was collected and loaded onto a 5 ml HisTrap FF crude column (Cytiva, Uppsala, Sweden). The column was washed with 50 mM Tris–HCl pH 8.4, 300 mM NaCl and 40 mM imidazole, and the protein was eluted with 50 mM Tris–HCl 8.4, 300 mM NaCl and 250 mM imidazole. Peak fractions were pooled, concentrated and loaded onto a Hiload 16 600 Superdex 200 prep grade column (Cytiva, Uppsala, Sweden) in 50 mM Tris–HCl pH 7.2 and 300 mM NaCl. Fractions containing full-length SARS-CoV-2 nsp14 were pooled, concentrated to 8.4 mg ml^−1^ and stored at −80 °C.

### Thermal shift assays for nsp10–fragment complexes using nanoDSF

Nsp10 in buffer (50 mM Tris–HCl pH 8.0 and 150 mM NaCl) and fragments in 100% DMSO were mixed to a final volume of 15 μL to yield a final concentration of 15 μM nsp10, 5 mM fragment and 6.7% (v/v) DMSO. Nsp10 without fragments was measured at the same DMSO concentration. The samples were loaded into a Prometheus NT.48 (Nanotemper, München, Germany) and measured using standard-grade capillaries. The samples were heated at 1 °C min^−1^, from 20 to 95 °C, and the unfolding of the protein was analysed according to the ratio of the wavelengths measured at 350 and 330 nm (tryptophan/tyrosine shifts) and with a laser power of 20%. From the resulting curves, the thermal unfolding transition midpoint *T*_m_ (°C), at which half of the protein population is unfolded, could be extracted.

### XFS with Nsp10

Crystallization of nsp10 has been reported previously.^12^ Nsp10 was concentrated to 69 mg ml^−1^ prior to crystallization. Crystallization was performed with a Mosquito (TTP Labtech, Melbourn, UK) crystallization robot for droplets with a final volume of 300 nL against a reservoir volume of 40 μl. The reservoir solution was selected from an optimization screen designed from an initial hit:^12^ 0.1 M Bis–Tris pH 6.7 and 2.4 M NaCl. Identical plates were prepared using a Dragonfly (TTP Labtech, Melbourne, UK). SWISSCI 96-well 3-drop plates (SWISSCI AG, Zug, Switzerland) plates were used, and three different 300 nL drops were set up using different protein concentrations (49, 59 and 69 mg ml^−1^) but with the same protein/precipitant ratio of 2 : 1. The plates were sealed and incubated at 20 °C. Crystals nucleated within a day and grew up to 100–150 μm in size over the course of 7 to 10 days.

### Nsp10 crystal soaking

For native crystals, a solution constituted of 0.1 M Bis–Tris pH 6.7, 2.4 M NaCl, 5.7% (v/v) DMSO and 17% (v/v) glycerol was prepared. Crystal soaking was performed by transfer of 0.7 μL of the soaking solution to a nsp10 crystallization drop, followed by equilibration of the re-sealed drop for 1 h at 20 °C. Then the crystals were harvested and cryocooled in liquid nitrogen. The fragment library was dispensed as 100 nL of 0.5–1 M DMSO stock solutions into the sub-wells of the SWISSCI 96-well 3-drop plate. Compounds were diluted in the subwells using a Mosquito crystallization robot with 1.6 μL of 0.106 M Bis–Tris pH 6.7, 2.54 M NaCl and 18% (v/v) glycerol. For fragment soaking, 0.7 μL of solubilized fragment was transferred to the crystallization drop with nsp10 crystals using the Crystal Shifter instrument (Oxford Lab Technologies, Oxford, UK) with a parallel transfer workflow, implemented in its control software.^20^ Final soaking conditions were 0.1 M Bis–Tris pH 6.7, 2.4 M NaCl, 15% (v/v) glycerol, 5% (v/v) DMSO and 25–50 mM fragment. Crystals were soaked for 2 h at 20 °C, then harvested with the assistance of the Crystal Shifter and cryocooled in liquid nitrogen prior to data X-ray diffraction data collection.

### X-Ray diffraction data collection, structure determination and refinement

Crystals were tested at the BioMAX beamline of MAX IV laboratory.^21^ Diffraction data were collected at 100 K as 90–180° rotation datasets. Analysis of the data was performed using the MAX IV computing cluster, with various combinations of available on-site data processing and automatic refinement pipelines, as implemented in the FragMAXapp software,^13,22^ using the nsp10 apo structure 6ZPE as a starting model. Electron density maps were examined manually in FragMAXapp through UglyMol viewer,^23^ and data sets with unexplained difference density peaks were subjected to further analysis. Additionally, PanDDA^14^ analysis was performed to identify potential low-occupancy ligands. Data sets of interest were processed with the autoPROC pipeline^24^ which uses XDS^25^ for data integration and AIMLESS^26^ for scaling. Resolution cut-off criteria were CC_1/2_ > 0.5 and mean *I*/sigma(*I*) > 1.3. Molecular replacement was performed with Phaser MR^27^ using 6ZPE as the search model. Ligand dictionaries and restraints were generated using AceDRG,^28^ model building was performed using *Coot*^29^ with iterative refinement with Refmac5^30^ and phenix.refine.^31^*mF*_o_ − *DF*_c_ omit maps were calculated in Phenix^31^ and visually inspected in *Coot*.^29^ The final models and corresponding structure factors were deposited in the Protein Data Bank (PDB) under accession numbers 7ORR, 7ORU, 7ORV, 7ORW. Diffraction images are available at the Integrated Resource for Reproducibility in Macromolecular Crystallography^32^ at http://proteindiffraction.org. All data from the crystallographic fragment screen can be accessed through the ZENODO data repository at DOI: https://zenodo.org under 10.5281/zenodo.5234009.

### MST to determine *K*_d_ values of fragment hits

His-SUMO-tagged nsp10 was diluted to 400 nM in MST buffer (50 mM NaPO_4_, pH 7.5, 150 mM NaCl and 0.05% Tween-20 (PBS-T)) with 0.1% PEG8000 and mixed with an equal volume of 100 nM RED-tris-NTA 2nd Generation labelling dye (Nanotemper, München, Germany). The mixed sample was incubated at room temperature for 30 min and then centrifuged at 15 000 × *g* for 10 min at 4 °C to remove any aggregates. The labelling quality was evaluated by the Pretest mode in MO.Control software (Nanotemper, München, Germany). Subsequently, *K*_d_ values for nsp10–fragment complex were determined as follows.

Fragments VT00221, VT00239 and VT00265 were serial diluted in nsp10 MST buffer for 15 rounds from 10 mM while VT00022 was serial diluted from 20 mM, with a dilution factor of 2. Each sample was mixed with an equal volume of 20 nM labelled nsp10 and incubated at room temperature for 20 min, then loaded into Monolith standard capillaries (NanoTemper Technologies). Samples were measured in a Monolith NT. 115 instrument (Nanotemper, München, Germany). The Pico-RED channel was used with 20% excitation power and 40% MST power. The temperature control was set at 25 °C. Measurements were controlled by the Binding Affinity mode in MO.Control software and the data were analysed in MO. Affinity Analysis software (Nanotemper, München, Germany).

For Fragment VT00265, a further ECP test (EDTA/Control peptide test) was carried out to verify ligand-induced fluorescence change caused by the fragment. For EDTA test, samples from three highest fragment concentrations and three lowest concentrations were centrifuged at 15 000 × *g* for 15 min at 4 °C. 7 μl from each sample was mixed with 7 μl of 50 mM EDTA, pH 7.4, then incubated at 37 °C for 30 min to remove labelling dye from His-tag. For control peptide test, 100 nM control peptide (Nanotemper, München, Germany) was incubated with an equal volume of 50 nM RED-tris-NTA 2nd Generation labelling dye at room temperature for 30 min. For the peptide-only sample, labelled control peptide was mixed with equal volume of ligand buffer (MST buffer with 10% dDMSO). For the peptide-ligand sample, labelled control peptide was mixed with an equal volume of 20 mM VT00265. All samples from EDTA and control peptide tests were loaded into Monolith standard capillaries and measured by the Expert mode in MO.Control software, with 20% excitation power and 40% MST power.

### Determination of the binding between nsp14 and nsp10 by MST

We optimised the buffer conditions for each MST experiment:

His-SUMO-tagged nsp14 ExoN was diluted to 800 nM with PBS-T buffer and subsequently labelled by mixing with an equal volume of 100 nM RED-tris-NAT labelling dye. The labelled protein solution was incubated on ice for 1 h. Serial dilution of nsp10 was prepared with a concentration rage of nsp10 from 4 mM to 120 nM in 50 mM Tris–HCl pH 8.0 and 150 mM NaCl. An equal volume of 20 nM labelled nsp14 ExoN in PBS-T was added to each diluted sample.

His-SUMO-tagged purified N7-MTase and Red-tris-NTA dye were diluted in assay buffer (60 mM HEPES pH 7.4, 150 mM NaCl and 0.1% PEG-8000) to 800 nM and 100 nM, respectively. 50 μl nsp14 N7-MTase was incubated with 50 μl dye on ice for 1 h. The labelled N7-MTase was mixed with nsp10. Nsp10 was diluted to final concentrations of 2–6 × 10^−5^ M by 2 fold serial dilution in ligand buffer (50 mM Tris–HCl pH 8.0 and 150 mM NaCl). The final concentration of N7-MTase in the assay was 10 nM. The mixed samples were incubated on ice for 1 h.

His-SUMO-tagged full-length nsp14 and RED-tris-NTA labelling dye were diluted with PBS-T buffer to 800 nM and 100 nM respectively. Diluted full-length nsp14 was labelled by adding the same volume of diluted labelling dye and incubating the mixture on ice for 1 h. To measure the *K*_d_ of the nsp14–nsp10 interaction, nsp10 was serial diluted in ligand buffer (50 mM Tris–HCl, pH 8.0, 150 mM NaCl) for 15 rounds from 1.9 mM, with a dilution factor of 2. Equal volumes of 20 nM full-length nsp14 was then mixed with nsp10. The mixed samples were incubated on ice for 1 h. To measure the *K*_d_ of the nsp14–nsp10 interaction in the presence of 3% deuterated DMSO (dDMSO), dDMSO was added to nsp10 and ligand buffer to a final concentration of 6%. Nsp10 was serial diluted in ligand buffer (50 mM Tris, pH 8.0, 150 mM NaCl, 6% dDMSO) from 1.9 mM, with a dilution factor of 2. Equal volumes of 20 nM full-length nsp14 was then mixed with each nsp10 sample, resulted in a final dDMSO concentration of 3%. Samples were incubated on ice for 1 h prior to the measurements.

All measurements were conducted using the Monolith NT.115 instrument. The data was analysed using MO.Control and MO.Affinity Analysis software.

### Calculation of physico-chemical properties and preparation of figures

The molecular weight, the clogP and the polar surface areas of fragment hits were calculated from their chemical structures using ChemDraw version 19.1. Hydrogen-bond donors and acceptors as well as MolLogS were calculated on the drug likeness prediction server of Molsoft: http://molsoft.com/mprop/. Figures were prepared using ChemDraw 19.2, Pymol (The PyMOL Molecular Graphics System, Version 2.4.1, Schrödinger, LLC) and Ligplot+.^16^ The default cut-off values in Ligplot+ were employed to identify hydrogen bond and hydrophobic interactions between residues of nsp10 and fragments.

## Abbreviations

CoV: Coronavirus
DTT: Dithiothreitol
EDTA: Ethylenediaminetetraacetic acid
ExoN: 3′-to-5′ exoribonuclease
IPTG: Isopropyl-β-d-thiogalactopyranoside
MTase: Methyltransferase
NaPO_4_: Sodium phosphate buffer
nsp10: non-structural protein 10
ORF: open reading frame
PMSF: Phenylmethylsulfonylfluorid
SDS–PAGE: sodium dodecyl sulphate–polyacrylamide gel electrophoresis
SARS: Severe Acute Respiratory Syndrome.

## Author contributions

Conceptualization: F. K., Z. F., W. K.; formal analysis: F. K., C. S., V. T., J. L., D. D., Q. W., X. S., M. N., A. R., Z. F., W. K.; funding acquisition: F. K., T. K., Z. F., W. K.; investigation: C. S., V. T., J. L., D. D., Q. W., X. S., M. N., A. R., Z. F., W. K.; project administration F. K., Z. F., W. K.; resources: F. K., V. T., T. K., Z. F., W. K.; supervision: F. K., Z. F., W. K.; validation: F. K., V. T., Z. F., W. K.; visualisation: F. K., Z. F., W. K.; writing – original draft: F. K., Z. F., W. K.; writing – review & editing: F. K., C. S., V. T., J. L., D. D., Q. W., X. S., M. N., A. R., T. K., Z. F., W. K.

## Conflicts of interest

The authors declare no conflict of interest.

## Supplementary Material

CB-003-D1CB00135C-s001

CB-003-D1CB00135C-s002

CB-003-D1CB00135C-s003

CB-003-D1CB00135C-s004

CB-003-D1CB00135C-s005

CB-003-D1CB00135C-s006

CB-003-D1CB00135C-s007

CB-003-D1CB00135C-s008

CB-003-D1CB00135C-s009

CB-003-D1CB00135C-s010

CB-003-D1CB00135C-s011

CB-003-D1CB00135C-s012
