# Supplementary material for: Identification of fragments binding to SARS-CoV-2 nsp10 reveals ligand-binding sites in conserved interfaces between nsp10 and nsp14/nsp16
Source: RSC Chem Biol. 2021 Oct 6;3(1):44–55. doi: 10.1039/d1cb00135c (PMC8729259; doi:10.1039/d1cb00135c)
Supplement: CB-003-D1CB00135C-s012 [file CB-003-D1CB00135C-s012.pdf]

Electronic Supplementary Material (ESI) for RSC Chemical Biology.
This journal is © The Royal Society of Chemistry 2021

Supplementary Information

Identification of fragments binding to SARS-CoV-2 nsp10 reveals ligand-binding sites in conserved interfaces between nsp10 and nsp14/nsp16.

Frank Kozielski[1], Céleste Sele[2], Vladimir O. Talibov[3], Jiaqi Lou[1], Danni Dong[1], Qian Wang[1], Xinyue Shi[1], Maria Nyblom[2], Annika Rogstam[2], Tobias Krojer[3], Zoë Fisher[2,4] & Wolfgang Knecht[2]

[1]School of Pharmacy, University College London, 29-39 Brunswick Square, London WC1N 1AX, UK.
[2]Department of Biology & Lund Protein Production Platform, Lund University, Sölvegatan 35, 22362 Lund, Sweden.
[3]BioMAX beamline, MAX IV Laboratory, Fotongatan 2, 22484 Lund, Sweden.
[4]Scientific Activities Division, Science Directorate, European Spallation Source, Partikelgatan 2, 22484 Lund, Sweden.

Corresponding author:
Wolfgang Knecht, Department of Biology & Lund Protein Production Platform, Lund University, Sölvegatan 35, 22362 Lund, Sweden, e-mail: wolfgang.knecht@biol.lu.se; tel. +46 46 2227785
Zoë Fisher, Scientific Activities Division, Science Directorate, European Spallation Source, Partikelgatan 2, 22484 Lund, Sweden, e-mail: zoe.fisher@ess.eu; tel. +46 721792250

Table S1. List of fragments from the FragMAX library used in nsp10 nanoDSF experiments. The T_m values of nsp10 in both nanoDSF experiments are given (empty in case atypical curves were obtained). The average T_m of nsp10 without any ligand under the same assay conditions was determined to be 46.6 ± 0.4°C (n = 11). The two fragments that showed a T_m equal or above the cut-off of 47.8°C are marked in yellow. The four fragments that were not tested in XFS are marked in light blue. The four fragments that were co-crystallised with nsp10 are marked in light green.

Fragment ID	SMILE	T_m°C (run 1)	T_m°C (run 2)
VT00019	O=S1(=O)CC(CN1)C1=CC=CC=C1	45.1	45.1
VT00022	N1C=NC(=C1)C1=CC=CC=C1		
VT00025	OC1CN(C1)C(=O)C1=CC=CC=C1	46.4	46.6
VT00029	OC(=O)CN1CCCCCC1=O	47.6	48.0
VT00033	CC1=C(C=CC(N)=C1)C(N)=O	45.7	46.3
VT00034	NC1=CC2=C(CCOC2)N=C1		
VT00036	CC(=O)NC1CCNCC1	46.3	46.3
VT00038	O=C1NC=C(C=C1)S(=O)(=O)N1CCCC1	45.8	45.5
VT00048	ClC1=CC=C2NC(=O)OC2=C1		
VT00049	NC1=CC=NN1CC1=CC=NC=C1	46.0	46.7
VT00056	NC1=NC(=CO1)C(F)(F)F	45.8	44.2
VT00058	COC1=CC=NC(=C1)C(N)=O	45.8	46.4
VT00059	CNC(=O)C1=CC=CS1	37.6	33.8
VT00064	COC1=CC(N)=CC(F)=C1	47.4	43.1

VT00065	CNCC1=NC=CS1	46.2	45.5
VT00066	CN1C=CN=C1C(O)C(F)(F)F	45.8	46.2
VT00068	CC1=NC=C(N1)C(F)(F)F	45.8	46.4
VT00077	CC1=CSC(CC(N)=O)=N1	46.4	46.7
VT00079	COC1=C(C=CC=C1)C(N)=O		
VT00082	OC(=O)C1=CC2=C(NC(=O)C2)C=C1	45.3	44.7
VT00083	CC1=CC=CN2C(N)=CN=C12	40.8	40.6
VT00084	CC1=CC=C(C=C1)S(=O)(=O)N1CCC[C@H]1C(O)=O	47.2	47.7
VT00085	NC1=CC=CC2=NC=CC=C12	45.8	45.2
VT00086	NC(=O)C1CCC2=CC=CC=C2N1	44.4	43.9
VT00087	NC1=NC(=CN=C1)N1CCCC1	38.5	34.2
VT00089	NC(=O)C1=CC2=C(C=NN2)C=C1	41.5	42.2
VT00096	NC1=CC(=NO1)C1CC1	45.5	46.7
VT00113	NC1=CN=C(N=C1)N1CCOCC1	42.0	39.5
VT00118	COC1=CC=CN=C1N		
VT00122	O=C1CC(CN1)C1CC1	45.8	46.6
VT00123	O=C1CC(CN1)C1=CC=NC=C1	46.0	46.0
VT00127	O=C1NN=CN1C1=CC=CC=C1	46.1	46.4
VT00128	OC1=CC=CC(=N1)C(F)(F)F	44.3	
VT00143	NC1=CC(=CC=N1)C#N	46.3	45.8
VT00147	CC(=O)N1CCCCC1CO	46.8	46.1
VT00150	COC1=CC=C(C=N1)C#N	42.3	42.6
VT00154	NCC1=CN2C=CC=CC2=N1		
VT00155	O=C1CCC2=CC=CC3=C2N1CC3	45.2	45.2
VT00159	O1N=CC=C1C1=CC=CC=C1	43.5	42.5
VT00162	N1C2=C(C=CC=C2)N=C1C1=CC=CN=C1	40.9	42.0
VT00164	NC(C1=CC=CN1)C(F)(F)F	47.1	47.2
VT00165	NCC1=CC2=C(CCC2)S1	46.5	46.1
VT00167	NC(=O)C1=C(C=CS1)C1=CC=CC=C1	44.5	44.9
VT00169	CC1=CN2C=C(N)C=CC2=N1	41.5	38.6
VT00172	N#CC1=C2C=CNC2=NC=C1	44.7	45.2
VT00173	NS(=O)(=O)C1=CN=CC=C1	44.4	45.0
VT00175	O=C1NC2=CC=CC=C2C=C1	44.4	44.7
VT00178	NC1CCN(C1=O)C1=CC=CC=C1	45.5	46.0
VT00180	NC(=O)C1=CNC2=CC=CC=C12	42.2	43.2
VT00181	CCS(=O)(=O)NC1=CC=CN=C1	44,2	44,8
VT00188	FC(F)(F)C1=CC(=O)NC=C1	43,1	43,6
VT00190	CC1=NC2=CC=CC=C2N1CCO		
VT00191	COC(=O)C1=C2C=CC=CN2C=N1	43,6	43,5
VT00193	CS(=O)(=O)N1CCOCC1	46,1	46,6
VT00198	CNC(=O)C1=CC=CC=C1	45,9	46,4
VT00204	COC(=O)C1CN(C)C(=O)C1	47.1	45.8
VT00207	COC(=O)CN1C=CC(N)=N1	46.1	46.5
VT00210	CN1CCCCS1(=O)=O	46.9	46.0
VT00211	OCC1CCCCO1	46.8	45.7
VT00212	CC(=O)CC1=NC(=NO1)C1=CC=C(C)C=C1	45.5	46.3

VT00213	CCC1=NOC(C1)C(O)=O	48.0	48.3
VT00215	COC(=O)C1=C(C)NN=C1C	46.0	46.0
VT00216	CN1C=CC=C1C(N)=O	45.3	45.7
VT00217	O=C1OCCC2=CC=CC=C12	44.6	43.7
VT00218	C1CCC2=NN=CN2C1	45.5	46.5
VT00219	CN(CC1=CC=CC=C1)C(N)=O	46.0	46.0
VT00220	CN1N=C(C=C1C(N)=O)C(F)(F)F	45.7	46.3
VT00221	NC1=CC=C2C=CC=CC2=N1	44.0	44.3
VT00222	CN(C(C)=O)C1=CC=CC=N1	45.9	44.5
VT00223	CN(C)S(=O)(=O)C1=CC=CC=C1F	45.0	42.7
VT00224	NC(=O)C1=CC=NC(Cl)=C1	46.0	45.7
VT00225	CC1=NNC(=C1)C(F)(F)F	45.5	46.3
VT00226	NC1=CC=C(C=N1)C(F)(F)F		
VT00227	NC1=NC(=CO1)C1=CC=CC=C1	44.0	42.9
VT00228	COC1=NC=CC=C1C(O)=O	46.7	46.8
VT00229	OCC1=CN(CC2=CC=CC=C2)N=N1	46.1	46.1
VT00230	NC1=C(C=NN1)C1=CC=C(F)C=C1	46.2	46.2
VT00231	NCC1=CC=CN=C1N1CCCC1	45.5	44.8
VT00232	C1=CN(N=C1)C1=CC=CC=N1		52.9
VT00233	CNC1=NC=CC=C1OC		
VT00234	OCC1=CN=C(N1)C1=CC=CC=C1		43.2
VT00235	CC1=CC=C(Cl)N=N1	46.3	46.4
VT00236	COC(=O)C1=CC=NN=C1	46.1	45.1
VT00237	CC(O)C1=NC(C)=CS1	47.2	46.2
VT00238	OC1=NOC2=CC=CC=C12	46.1	46.2
VT00239	CN(C)C1=CC=NC(N)=C1	43.0	43.1
VT00240	N1C=CC2=CC=CN=C12	42.4	43.3
VT00241	OC1=CC=C(Cl)N=C1	44.5	
VT00242	CC1=CC(C)=NC=N1	46.8	45.9
VT00243	CN1C=NC2=C(Cl)N=CN=C12		
VT00245	CC1=CC=C(S1)C1=CC(=NN1)C(O)=O	44.2	44.3
VT00246	C1=NN2C=CC=CC2=N1	42.3	42.7
VT00247	CC1=NN2CCC(=O)NC2=C1	46.1	46.3
VT00248	CS(=O)(=O)N1C=CC=C1	45.7	46.3
VT00249	O=C1N[C@@H](CO1)C1=CC=CC=C1	45.6	46.1
VT00250	O=C1NC2CCC1CC2	45.9	46.3
VT00251	O=C1CNCCN1C1=CC=CC=C1	46.7	46.8
VT00252	O=C1NCCN2CCCC12	46.2	45.9
VT00253	CC1CCC2(CC(=O)N2)CC1	45.7	46.0
VT00254	O=C1NC(=CS1)C1=CC=CN=C1	45.7	45.6
VT00257	CNS(=O)(=O)C1=C(C)ON=C1C	45.7	46.3
VT00258	COC1=C2NN=CC2=CC=C1		
VT00259	COC(=O)C1=NNC2=C1CCCC2	45.6	48.1
VT00260	CCS(=O)(=O)N1CCCC(C1)C(O)=O	47.6	47.8
VT00261	N#CC1=CC=CN=C1N1CCOCC1	44.7	45.3
VT00263	CN1CCCN(C)C1=O	40.2	46.2
VT00264	COC1=CC=C2NC(C)=NC2=C1		

VT00265	NC1=C2N=CNC2=CC=C1	46.6	45.3
VT00267	CN1CCCC1CO	45.3	45.3
VT00268	C1=CC=C(C=C1)C2=CSC(=N2)N	46.4	46.4

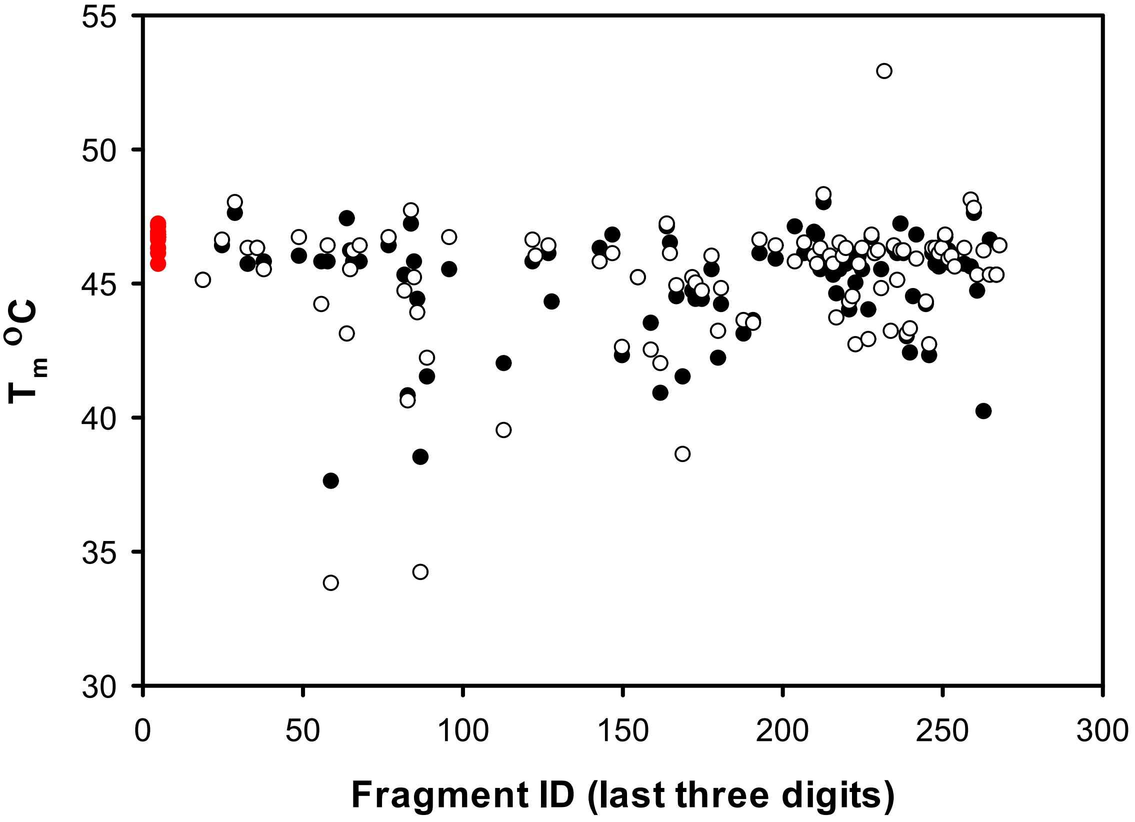

Figure S1. Thermal shift assays of nsp10 in the presence of fragments. Black circles are from the first experiment and empty circles from the second one. To the left, the T_m values of nsp10 without any ligand under the assay conditions (average: 46.6 ± 0.4°C (n=11)) are shown as red dots. Fragments that lead to atypical curves (Table S1) are not included in this plot.
